# Association of GSTO2 (N142D) Genetic Polymorphism and Acute Rejection of Liver

**Published:** 2016-08-01

**Authors:** M. Khosravi, I. Saadat, M. H. Karimi, S. A. Malek Hosseini

**Affiliations:** 1 Department of Biology, College of Sciences, Shiraz University, Shiraz, Iran; 2 *Transplant Research Center, Shiraz University of Medical Sciences, Namazi Hospital, Shiraz, Iran*

**Keywords:** Graft rejection, Glutathione S-Transferase, Polymorphism, genetic, transplantation, Metabolic Detoxication, Phase II

## Abstract

**Background:**

Acute rejection is the main problem in liver transplantation that occurs in the first days or months of transplantation. It includes histological and cellular rejection. Acute histological rejection is confirmed by biopsy. Glutathione S-transferase family is the most important genes in phase II detoxification working in xenobiotic and drug metabolism*. GSTO2 *is one of the members of this family*. GSTO2* (N142D) polymorphism may influence metabolism of immunosuppressive drugs.

**Objective:**

To determine if *GSTO2* polymorphism has association with acute liver rejection.

**Methods:**

The present study included 120 patients with histological-proven acute liver rejection and 182 patients without acute rejection. Both groups were matched for sex and age. To determine variants of *GSTO2, *we used polymerase chain reaction-restriction fragment length polymorphism (PCR-RFLP).

**Results:**

There was a significant association between the *GSTO2* genotype and acute liver rejection (NN: OR: 3.642, 95% CI: 1.179–5.444) and (ND: OR: 2.533, 95% CI: 1.672–8.149) compared to those with DD geneotype.

**Conclusion:**

Recipients with either NN or ND genotype for *GSTO2 *are more likely to develop acute liver rejection compared to those with DD genotype.

## INTRODUCTION

Liver transplantation is one the best treatments for patient suffering from end-stage liver disease [1]. Despite using immunosuppressive drugs and new methods, acute rejection (AR) is one of the main obstacles after liver transplantation. Although patient survival is increased to 80% in one year, incidence of acute liver rejection (ALR) is still 40%–80% [1]. ALR is an immune process where lymphocytes play major roles in. Immunosuppressor drugs, such as cyclosporin are inhibited from calcineurin activity, so T cells cannot be activated and migrates towards transplanted organ and therefore, rejection does not occur [2].

Glutathione-S transferases (GST) are the main enzymes in phase II detoxification that play important role in xenobiotic, and drug metabolism, which catalyze binding of glutathione to these substrates causing them become more water soluble, so that they can be excreted through bile or urine [3]. Mutation in these genes can affect detoxification that may ultimately cause cancer and other diseases [4]. Omega class of the GST family, included *GSTO1* and *GSTO2*. *GSTO2* is on 10q24.3 and has six exons that spans over a 24.5-kbp region that lies 7.5 kbp downstream to *GSTO1*. *GSTO2 *has a Cys in its active site that plays an important role in arsenic detoxification. *GSTO2 *is expressed in whole the body organs, especially liver, kidney, skeletal muscles and prostate [5].

*GSTO2* (N142D) is one of the polymorphisms of this gene with A>G transition at nucleotide position 424 of exon 5. This change reduces the expression of this enzyme by 20% [6]. Some studies show that this polymorphism is associated with colorectal cancer [7], gastric cancer [8] and ovarian cancer [9]. There are evidence that these polymorphisms have also roles in asthma [10, 11] and liver carcinoma [12]. We know that GSTs family interferes with drug metabolism. Association of ALR and this polymorphism have so far not been examined. We therefore, conducted this study to determine whether *GSTO2* (N142D) can affect ALR.

## PATIENTS AND METHODS

Study Groups

In the current study, 302 recipients of liver transplants between 2007 and 2011, were studied. All of the patients were Iranian and transplanted at the Transplantation Center of Namazi Hospital affiliated to Shiraz University of Medical Sciences, Shiraz, southern Iran. The study protocol was approved by Ethics Committees of Shiraz University and Shiraz University of Medical Sciences. In the recipients, we investigated the graft outcome and AR episode(s) for at least six months. Acute rejection episodes were identified by an expert gastroenterologist team based on the accepted criteria such as increased serum liver enzymes levels and total serum bilirubin level in the absence of biliary problems, histological findings after biopsy of the liver and clinical and biochemical response to high-doses of steroids, according to criteria for AR described by Banff schema [13]. The routine immunosuppression regimen consisted of tacrolimus or cyclosporine with mycophenolate mofetil and steroids. Drug dosage was adjusted to maintain a target therapeutic blood level of 200 ng/mL for CsA (5 mg/kg/day) or 2–3 mg twice a day for tacrolimus. Methylprednisolone was added to immunosuppressive regimen for the patient with signs or symptoms of rejection. The patients were divided into two groups according to presence (AR group) or absence (non-AR group) of acute rejection. Donors were selected on the basis of ABO blood group compatibility. HLA matching is routinely not checked for liver transplantation in our center.

DNA Extraction

The buffy coat of the whole blood from liver transplanted patients was available in the sample bank of Transplant Research Center. Patient’s DNA was extracted with DNP kit (CinnaGen, Iran) according to the manufacturer’s instructions.

Genotyping

**Figure 1:**
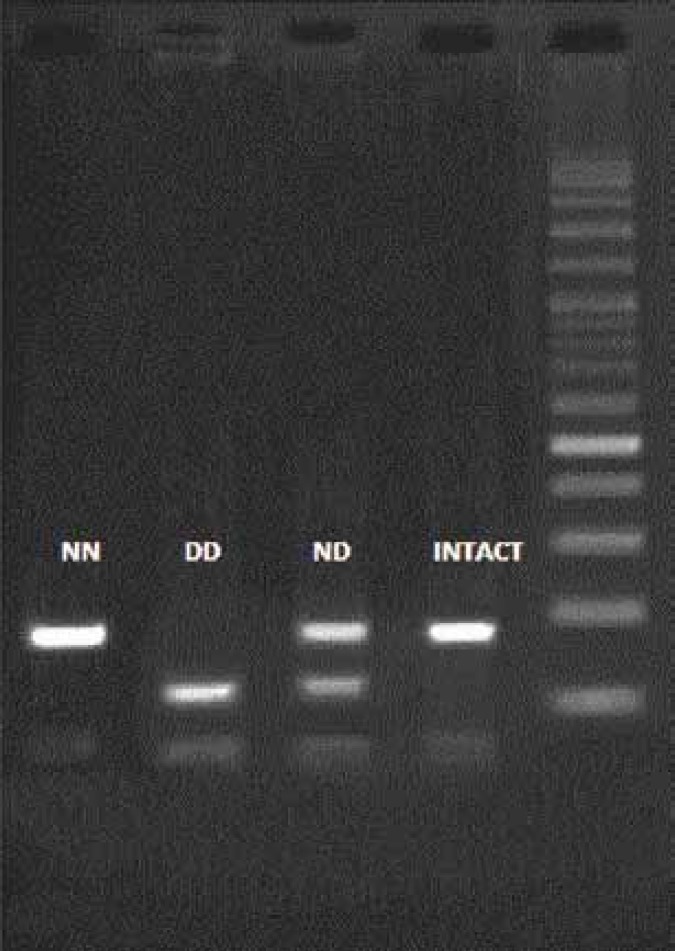
Genotyping of the *GSTO2* (N142D) polymorphism by MboI RFLP. From right to left the lanes are DNA size marker (100-bp ladder), intact (185-bp), ND genotype (185-bp, 122-bp, and 63-bp), DD genotype (122-bp and 63-bp), and NN genotype (185-bp), respectively

Patients’ genotypes were determined using polymerase chain reaction-restriction fragment length polymorphism (PCR-RFLP). PCR mixture included MgCl_2 _(0.45 mM), KCl (50 mM), Tris-HCl (10 mM, pH of 8.3), dNTP (0.2 mM), each primer (4 μM), Taq DNA polymerase (1 U) (all from CinnaGen, Iran) and DNA sample. Sequences of forward and reveres primers were 5’-AGGCAGAACAGGAACTGGAA-3’ and 3’-GAGGGACCCCTTTTTGTACC-5’, respectively. The PCR program consisted of an initial pretreatment at 95 °C for 5 min, with 35 cycles fallowed by denaturation at 95 °C for 1 min, annealing at 65 °C for 40 sec and extension at72 °C for 1 min. Then, 2.5 μL Tango buffer and 5 U MboI enzyme added to each PCR product tube and incubated at 37 °C for 14 hours. Then, loaded in 2% agarose gel and stained by ethidium bromide and finally illustrated by UV translluminator. When our genotype was *GSTO2 *NN, we observed one fragment (185 bp) in the gel; in heterozygote case (ND), three fragments (185-bp, 122-bp, and 63-bp) and in DD homozygote two fragments (122-bp, and 63-bp) was observed (Fig 1).

Statistical Analysis

We use SPSS^®^ for Windows^®^ ver 17.0 (SPSS Inc, Chicago, IL, USA) for data analysis. We used *Student’s t *test for continuous variable. χ^2^ test was used for categorical variables and logistic binary regression test for risk of liver problems.

## RESULT

A χ^2^ test was performed for *GSTO2* polymorphism to determine if the distribution followed the Hardy-Weinberg equilibrium. Regardless of rejection, the studied gene was in Hardy-Weinberg equilibrium (P>0.05). From 302 patients, 120 had AR (mean±SD age of 30.9±18.3 years) and 182 patients did not (mean±SD age of 29.1±19 years). The frequency of D allele was 35% in patients with ALR and 47.3% in those without ALR. There was a significant relationship between *GSTO2 *(N142D) polymorphism and ALR; increase in D allele reduced the likelihood of ALR; there was a significant association between *GSTO2* genotypes and ALR (ND genotype: OR=2.533, p=0.002; NN genotype: OR=3.642, p=0.007) and also for alleles (OR=1.66, p=0.002) (Table 1). Patients with ALR included 44 females and 76 males; those without ALR included 66 females and 116 males. There was no correlation between sex and ALR (OR=0.983, p=0.943). The mean±SD age of patients with ALR was 30.9±18.3 and those without ALR was 29.1±19 years; the age did not affect the likelihood of developing ALR (OR=1.27, p=0.943). Nor was association between the underlying disease and ALR (hepatitis C: OR=1.776, p=0.313; hepatitis B: OR=1.065, p=0.301). Origin of liver, living donor or cadaver, was also not a risk for ALR (OR=1.295, p=0.38).

**Table 1:** Association of *GSTO2* (N142D) genetic polymorphism and liver acute rejection

	With ALR (%)	Without ALR (%)	OR (95% CI)
Genotype			
DD	10 (8.3)	38 (21)	1
ND	64 (53.3)	96 (52.7)	2.533 (1.627–8.149)
NN	46 (38.3)	48 (26.3)	3.642 (1.179–5.44)
Allele			
D	84 (35)	172 (47.3)	1
N	156 (65)	192 (52.7)	1.66 (1.17–2.36)

## DISCUSSION

AR is one of the main obstacles after liver transplantation. Several studies show that an increase in D allele frequency decreases the risk of colorectal and gastric cancer [7, 8]. It has been shown that many polymorphisms in the immune system can affect ALR [14-18]. In 2011, it was shown that there is no association between *GSTM1* and *GSTT1* with ALR [19]. Another study conducted in 2013 revealed that recipient/donor* GSTT1* genotype mismatch (present/null or null/present) is significantly associated with development of acute cellular rejection [20], however, the association of *GSTO2 *and also this polymorphism have so far not been examined. In the current study, we showed that increasing frequency of D alleles decreased the risk ALR. The NN genotype is associated with elimination of immunosuppressive drugs faster than the ND, DD genotypes; ND genotype is faster than DD genotype too. Therefore, the risk of ALR is increased with presence of N allele. In several studies it was shown that concentration of liver enzymes can be useful for predicting ALR [21]. We found that a serum AST level measured on the third day of transplantation, >300 U/L was associated with an increased risk of ALR.

In 2013, we showed that patients with D allele of *GSTO2* are more prone to develop hepatic failure. We also observed that males are more prone to develop hepatic failure leading to liver transplantation [22].

A recent study conducted on a group of Iranian people indicated that *GSTO2* polymorphism N142D is not associated with acute renal rejection [23]. An important point was that the combined effect of using a cadaveric kidney transplant and presence of *GSTO2* DD genotype in the recipient, results in significant increase in risk of acute rental rejection (OR=3.82, p=0.02). This reflects a probable reduction in function of DD genotypes, as anti-oxidant enzymes involved in phase II detoxification, with consequent increase in acute inflammatory processes [22].
